# Influence of Variable Radius of Cutting Head Trajectory on Quality of Cutting Kerf in the Abrasive Water Jet Process for Soda–Lime Glass

**DOI:** 10.3390/ma13194277

**Published:** 2020-09-25

**Authors:** Marzena Sutowska, Wojciech Kapłonek, Danil Yurievich Pimenov, Munish Kumar Gupta, Mozammel Mia, Shubham Sharma

**Affiliations:** 1 Department of Production Engineering, Faculty of Mechanical Engineering, Koszalin University of Technology, Racławicka 15-17, 75-620 Koszalin, Poland; marzena.sutowska@tu.koszalin.pl (M.S.); wojciech.kaplonek@tu.koszalin.pl (W.K.); 2 Department of Automated Mechanical Engineering, South Ural State University, Lenin Prosp. 76, 454080 Chelyabinsk, Russia; danil_u@rambler.ru (D.Y.P.); munishguptanit@gmail.com (M.K.G.); 3 Key Laboratory of High Efficiency and Clean Mechanical Manufacture, Ministry of Education, School of Mechanical Engineering, Shandong University, Jinan 250061, China; 4 Department of Mechanical Engineering, Imperial College London, Exhibition Rd., South Kensington, London SW7 2AZ, UK; 5 Department of Mechanical Engineering, IK Gujral Punjab Technical University, Jalandhar-Kapurthala Road, Kapurthala 144603, Punjab, India; shubham543sharma@gmail.com or

**Keywords:** abrasive water jet machining, cutting kerf, soda–lime glass, radius of the cutting head trajectory, quality

## Abstract

The main innovation of this article is the determination of the impact of curvature of a shape cut out in a brittle material using an abrasive water jet (AWJ) process as an important factor of the machined surfaces. The curvature of a shape, resulting from the size of the radius of the cutting head trajectory, is one of the key requirements necessary for ensuring the required surface quality of materials shaped by the abrasive water jet process, but very few studies have been carried out in this regard. An important goal of the experimental studies carried out here and presented in this work was to determine its influence on the quality of the inner and outer surfaces of the cutting kerf. This goal was accomplished by cutting the shape of a spiral in soda–lime glass. For such a shape, the effect of radius of the trajectory of the cutting head on selected parameters of the surface texture of the inner surface of the cutting kerf (IS) and the outer surface of the cutting kerf (OS) was studied. The obtained results of the experimental studies confirmed that the effect of the curvature of the cut shape is important from the point of view of the efficiency of the glass-based brittle material-cutting process using AWJ. Analyses of the surface textures of the areas located in the upper part of the inner and outer surfaces separated by the use of AWJ machining showed that the OS surfaces are characterized by worse technological quality compared with IS surfaces. Differences in the total height of surface irregularities (given by St amplitude parameter), determined on the basis of the obtained results of the measurements of both surfaces of the cutting kerf, were as follows: ΔSt_r = 50_ = 0.6 μm; ΔSt_r = 35_ = 1 μm; ΔSt_r = 15_ = 1.3 μm. The analysis of values measured in areas located in the more sensitive zone of influence of the AWJ outflow proved that the total height of irregularities (St) of the OS was higher. Differences in the total heights of irregularities for inner and outer surfaces of the cutting kerf were as follows: ΔSt_r = 50_ = 2.1 μm; ΔSt_r = 35_ = 3 μm; ΔSt_r = 15_ = 14.1 μm, respectively. The maximum difference in the total heights of irregularities (St), existing between the surfaces considered in a special case (radius 15 mm), was almost 20%, which should be a sufficient condition for planning cutting operations, so as to ensure the workpiece is shaped mainly by internal surfaces.

## 1. Introduction

**Figure 1:**
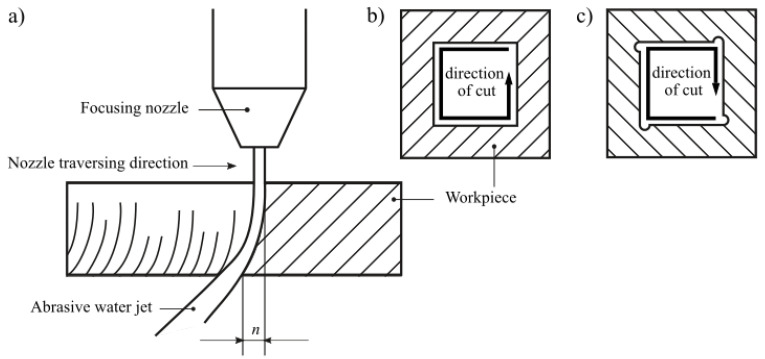
Phenomena occurring during material cutting with high-pressure abrasive water jet (AWJ), important in the context of the quality of this process—deviation of the AWJ: (**a**) graphical interpretation of the phenomenon; (**b**) workpiece top view; (**c**) workpiece bottom view with errors in the shape of corners caused by the jet lag.

Abrasive water jet (AWJ) machining is a nontraditional advanced hybrid method used for shaping a wide range of modern and conventional materials, which can replace other more traditional machining techniques, an aspect which was presented by Liu et al. [1]. As stated by Hashish [2], AWJ machining consists of shaping materials using a highly concentrated water jet doped with abrasive grains. The most commonly used abrasive is garnet [3]. In addition to numerous advantages, such as those mentioned by Krajcarz [4] (including no thermal distortion, high flexibility, high machining versatility, small machining force, and the absence of a heat-affected zone), this method has some limitations, as reported Wang et al. [5]. While cutting materials using AWJ, two phenomena may be observed which are important in the context of the quality of this process. The first is the deviation of the AWJ in the opposite direction to the movement of cutting head, as described by Hlaváč et al. [6]. This means that, during movement of the cutting head along the workpiece, the outflow of the jet occurs with a delay in relation to its site of entry into the material. This phenomenon is presented graphically in Figure 1a.

The distance between the entry and outflow points of the jet was defined by Hashish [7] as the jet lag. The shape that the jet adopts during the cutting process of materials is expressed on the side surfaces of the workpieces usually in the form of parallel striation whose intensity increases in the area of the lower part of the cutting zone [8]. During rectilinear cuts, the AWJ can move at high speed on the surface of the workpiece, as its deviation does not affect the accuracy of the process. However, in the case of the corners cut, an excessive speed of AWJ movement may cause the creation of shape errors, as reported by Chen et al. [9]. This situation is presented graphically in Figure 1b. For this reason, when cutting objects with complex shapes, the traverse speed must be properly selected to eliminate such kinds of technological errors. It should be emphasized that the phenomena discussed here can be practically eliminated by implementing appropriate algorithms in the controllers of devices intended for cutting materials employing AWJ. The AWJ systems currently used in industry enable carrying out the precise cutting process via the appropriate selection of its parameters, which means the above-discussed machining method may be treated as a good alternative to other methods of cutting materials.

**Figure 2:**
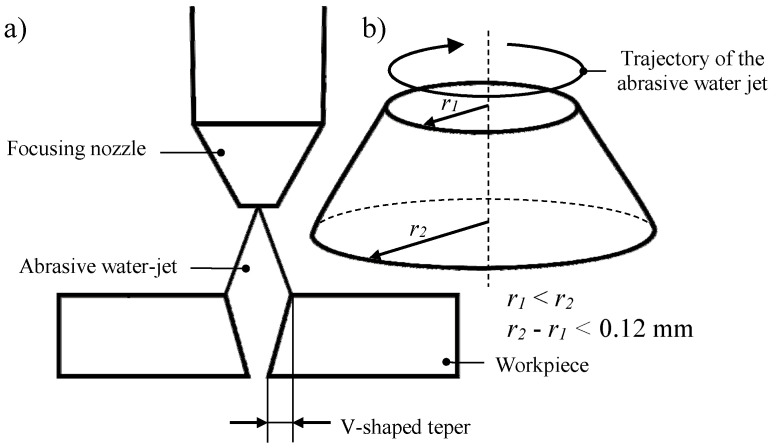
Phenomena occurring during material cutting with a high-pressure AWJ, important in context of the quality of this process: (**a**) formation of a V-shaped taper; (**b**) convergence effect.

The shape of the AWJ also significantly affects the outline of the cutting kerf, which changes in the area of the cutting zone, as shown by Wang et al. [10]. These differences in the width of the resulting kerf were defined by Hlaváč et al. [11] as a taper. This can be positive or negative, depending on whether the width of the cutting kerf reaches a larger size in the area of the entry or outflow jet from the material. In industrial practice, the width of the cutting kerf is usually smaller in the lower part of cutting zone, taking the shape of the letter V, as presented in Figure 2a.

The formation of a V-shaped taper during the AWJ cutting process, as shown by Kechagias et al. [12], illusively increases the radius of cutting curve while the cutting head moves along a circular path. This phenomenon is characterized by the differentiation of the diameter of the cut object in the area of AWJ entry and outflow points. This situation is presented graphically in Figure 2b. Higher differences between both diameters denote closer proximity of the value of the traverse speed to the maximum (critical) speed.

The above-described results of the effect of the shape of the jet on the outline of the cutting kerf are presented in Figure 2 on an exaggerated scale. In fact, with properly selected machining parameters, they are almost imperceptible. The results of the experimental studies carried out by Wu et al. [13] clearly indicate that the value of the V-shaped taper of the cutting surface, even in the case of not optimally selected process parameters, does not exceed 0.1 mm. Under normal conditions, this value is lower, which means that the method of machining materials being discussed may be considered as relatively accurate. Given the above, AWJ, which is a universal technological tool, provides itself with a wider range of machining applications, enabling the shaping of many types of materials, such as metals and its alloys [14,15,16,17,18,19], ceramic, glass [20], composites [21,22], flammable materials, leather, and natural stone-based materials [23], as well as regenerating the cutting ability of grinding tools [24]. Nevertheless, there is still a need for further research related to this technology in order to increase the efficiency of material machining. The obvious method is to generate a water jet with very high pressures reaching up to 700 MPa. An alternative solution to that presented above is eliminating the use of high pressures through the use of a pulsating flow of water jet. These were described in the literature by Hloch et al. [25] and Lehocka et al. [26].

The generation of vibrations, i.e., the vibration spectrum, is one of the accompanying features which indirectly characterizes the abrasive water jet cutting process, its quality, and its expected result. Therefore, it is possible and advisable to apply the analysis of accompanying physical phenomena and the determination of their limit values in the controlling phase of cutting processes through a simple control of at least one quantity which characterizes the process. These were presented in works by Hreha et al. [27].

The quality of surfaces cut with AWJ is assessed similarly to that in the case of other machining methods (for example, Matuszewski et al. [28] discussed the influence of the configuration of the geometric structure of the surface to be treated on the course of the wear process of friction pairs machined using various machining methods, and Bustillo et al. [29] solved the problem of predicting it with the help of artificial intelligence). First of all, indicators of dimensional and shape accuracy (as deviations from the nominal size) are determined. In addition, the shape of surface texture of the cut is also characterized. An important role in the assessment of the technological quality of the cutting process is played by the quality indicators of the shape of the cutting kerf, as well as the quality indicators of the topography and microgeometry of the surface cut. These were described in the literature by Borkowski et al. [30], as well as in the VDI 2906-5:1994 standard developed by the Association of German Engineers [31].

A wide range of two- and three-dimensional (2D and 3D) parameters can be used for a quantitative assessment of cut surface roughness. The most commonly used parameters for assessment of the surface texture in 2D, as presented in works by Löschner et al. [32], Zagórski et al. [8], Hreha et al. [33], and Klichova and Klich [34] and defined in the ISO 4287:1997 standard [35], are the amplitude parameters *Ra, Rq*, and *Rt.* The 3D parameters can be divided into the following groups: amplitude, area and volume, spatial, hybrid, and functional; they are included in the ISO 25178-2:2012 standard [36] and the EUR 15178 EN report [37]. The significant influence of cutting parameters (traverse speed, water pressure, abrasive feed rate) was observed in the case of amplitude parameters (especially *Sa*, *Sq*, *St*), as noted by Aich et al. [20] and Borkowski et al. [30]. The shape of the topography of the surfaces cut by AWJ also indicates the presence of waviness, as presented Sutowska [38], whose intensity increases with the distance from the jet’s input zone to the material. For a quantitative assessment of this phenomenon, a set of waviness parameters (especially *Wt, WSm*), defined in the ISO 4287:1997 standard [35], can be used. The general characteristics of above-mentioned roughness and waviness parameters are given in Table 1.

The basic parameters of the process that characterize the cutting of materials by the AWJ are as follows: water jet pressure *p*; traverse speed *v*; abrasive feed rate *m_a_*; water jet orifice diameter *d_o_*; focusing tube diameter *d_f_*; standoff distance *l* [40]. Knowledge of the effect of machining conditions on the quality of the obtained cuts ensures high-quality cutting.

The curvature of the shape cut out, resulting from the determined radius of the trajectory of the cutting head, is one of the essential conditions of the quality of the cut using AWJ. A good example which facilitates the analysis of the impact of this parameter on the quality of the cutting kerf is provided by a spiral. Its shape allows smoothly (continuously) assessing the changing curvature of the outline being cut out by an AWJ. This is advantageous and allows the assessment of the inner and outer surfaces (IS and OS), whose varying shapes may suggest what should be product and what should be waste, due to the level of dimensional deviations and the quality of the surface texture.

## 2. Methodology of Experimental Studies

### 2.1. Main Goal

The main goal was to investigate the influence of the radius of the cutting head trajectory on the surface quality of areas adjacent to the upper and lower cutting zone. Such differentiation refers to the shaping of the inner (IS) and outer (OS) surfaces of the cutting kerf. The innovativeness of the described studies is based on the fact that, although the curvature of the shape cut out by an AWJ (resulting from a fixed radius of the trajectory of cutting head) is one of the important conditions for the quality of materials cutting with AWJ, no studies have yet been carried out to determine its impact on the quality of the cuts obtained. In the sections below, details related to conditions in which the experimental studies were carried out, as well as results of the experiments, along with their analyses, are given.

### 2.2. Characteristics of the Samples

A planned cycle of experimental studies was carried out on soda–lime glass. The selection of this type of material was made deliberately as it is easier to expose the differences in the shape of the IS and the OS. This is due to the greater susceptibility of this brittle material to hydro-jetting erosion, as well as its limitations resulting from machining by the use of conventional machining techniques.

**Figure 3:**
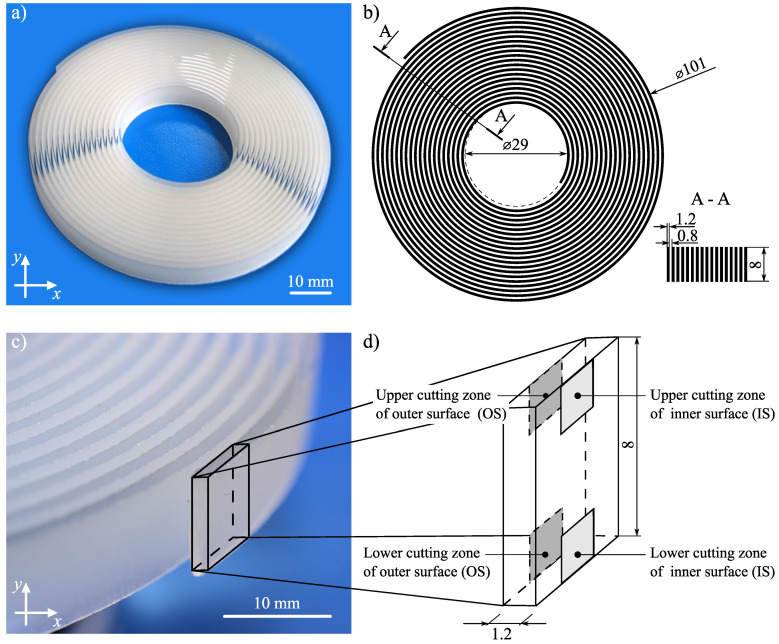
The soda–lime glass spiral used in the experimental studies: (**a**) general view of the spiral; (**b**) basic geometrical dimensions; (**c**,**d**) a section of the spiral with the upper/lower inner and outer surfaces (IS and OS) marked.

Soda–lime glass is an amorphous body, created as a result of supercooling molten raw minerals and other inorganic substances without the crystallization of ingredients. This relatively inexpensive and widely available glass is a base material for most types of glass (colorless, colored, and patterned). Its chemical composition and selected physical properties are given in Table 2, whereas a general view of the spiral used in the experimental studies is presented in Figure 3. The mass of the spiral was 30.74 g.

### 2.3. Conditions and Course of the AWJ Process

**Figure 4:**
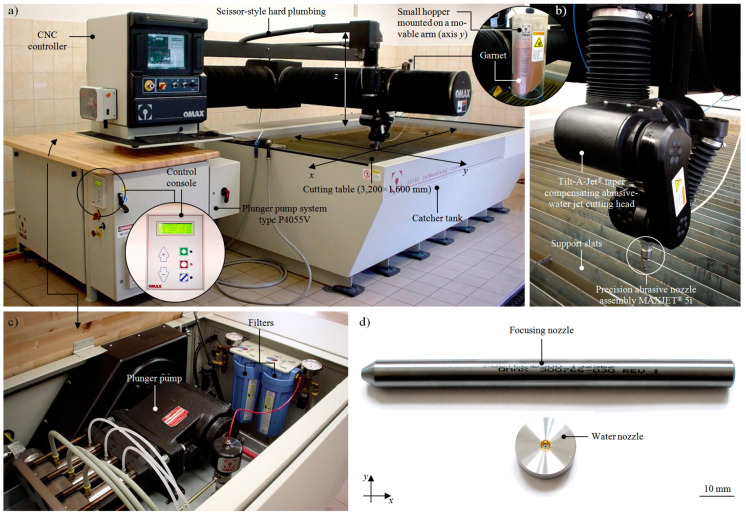
Precision AWJ system used during experimental studies—JetMachining^®^ Center type 55100 (OMAX Corp., Kent, WA, USA): (**a**) general view of the center with main components; (**b**) close-up on the Tilt-A-Jet^®^ cutting head (OMAX Corp., Kent, WA, USA) and abrasive nozzle assembly MAXJET^®^ 5; (**c**) close-up on the plunger pump and filters; (**d**) two type of nozzles used in the cutting head.

The cutting process was carried out using a JetMachining^®^ Center type 55100 cutting machine produced by OMAX (Kent, WA, USA). This precision AWJ system, whose general view is presented in Figure 4, is widely used in many modern applications, as presented by Zhao and Guo [41], Linke et al. [42], and Saurabh et al. [43].

The Jet Machining^®^ Center was equipped with a P4055V plunger pump (Figure 4c), driven by a 30 kW electric motor. Water drawn from the waterworks system was used to power the pump, which produces=d a water jet with the following parameters: *p_max_* = 385 MPa; *Q_max_* = 4.9 dm3/min. From the pump, water under high pressure was supplied by special tubes to the MAXJET^®^5 cutting head operating as part of the Tilt-A-Jet^®^ mechanism (Figure 4b). The cutting head body used a set of nozzles (Figure 4d). The center was equipped with an automatic abrasive feeder, used in connection with a small hopper mounted on a movable arm (axis *y*). This allowed the abrasive particles to be delivered to the cutting head continuously without interrupting the JetMachining^®^ Center. An important element of the AWJ system was a cutting table whose working range was 3200 × 1600 mm. Inside the table frame, a catcher tank was placed equipped with support slats on which the material to be cut was attached. Dedicated computer-aided design (CAD) software, namely, OMAX Layout, was used to prepare drawings of the outline of the cut object and to specify the cutting path, whereas OMAX Make software, installed on a controller equipped with a color monitor and an industrial keyboard, was used to start the cutting process. The controller was used to control the cutting head during the process, the intensity of the abrasive feed, and the water jet pressure. Additionally, the controller screen allows for continuous monitoring of the course of the process.

After the cutting process started, the geometry of the spiral and the cutting path of the sample was generated using the OMAX Layout software (OMAX Corp., Kent, WA, USA). Then, in the OMAX Make software, the cutting path created in OMAX Layout was opened. In specifying the cutting process conditions, the type and thickness of the processed material (plate glass soda lime silica, *g* = 8 mm) and the cut quality (Quality 1) were chosen. On this basis, the OMAX Make software calculated the value of the traverse speed *v* = 427 mm/min and the abrasive feed rate *m_a_ =* 0.363 kg/min (Garnet 80 mesh size). A water nozzle with a diameter of *d_o_* = 0.38 mm and focusing tube diameter *d_f_* = 0.76 mm was mounted in the cutting head body. Next, the material prepared for the cutting process in the form of a soda–lime glass square plate (150 × 150 × 8 mm) was placed upon the cutting table of the AWJ system. Due to the small size of the spiral, the plates were directly mounted on a special base (a waterjet brick), fixed to the cutting table. The last procedure before the cut process was to determine the working length of the AWJ, namely a distance between the outflow from the focusing nozzle and the material surface of *l* = 1.5 mm. After the cutting process was finished, the samples were thoroughly washed and dried using compressed air.

### 2.4. Characteristics of Measurement Systems and Course of Measurement Process

After finishing the AWJ cut process, the geometrical shape of both surfaces of the cutting kerf was measured using one of the advanced optical methods involving optical profilometry. In carrying out measurements, a Talysurf CLI 2000 multisensory optical profilometer (Taylor-Hobson, Leicester, Great Britain) was used. This instrument was extensively described by Kapłonek et al. [44], while its selected applications were presented by Yuan et al. [45], Nadolny et al. [46], and Fan [47]. Considering the relatively large variation in the heights of irregularities occurring on shaped surfaces due to the AWJ cutting process, measurement of their surface texture was carried out using a type LK-031 laser sensor (Keyence Corp., Osaka, Japan) [41] installed in the measuring head of the Talysurf CLI 2000.

The microstructure measurements of the surfaces cut by the AWJ were carried out using a Quanta 200 Mark II scanning electron microscope (SEM) (FEI Company, Hillsboro, OR, USA). This high0resolution environmental microscope (ESEM) is intended for the observation of samples at a magnification of 30 × to ~1,000,000 × in high or low vacuum and variable pressure conditions. During measurements, the low-vacuum mode (LowVac) for observing the surface was used. The SEM micrographs were acquired for a surface area of 2.133 × 1.966 mm with a magnification of 140 × at an accelerating voltage of *Ua* = 15–20 kV. Due to its broad observation and measurement capabilities, this microscope is used in many areas of modern science and technology. Examples of its use were given by Chen et al. [48], Nadolny et al. [49], and Kapłonek and Ungureanu [50].

The general characteristics of the observation/measurement systems used in the experimental studies are presented in Table 3.

## 3. Results and Discussion

The analysis of the results of the experimental studies was divided into the following phases:

1. A study of the influence of the curvature of the cut out shape on the IS and OS surface texture shaped using an AWJ, carried out on the basis of the calculated values of roughness and waviness parameters characteristic for this type of machining [24] measured by the Talysurf CLI 2000 multisensory optical profilometer (Taylor-Hobson, Leicester, Great Britain) (Section 3.1).
2. A study of the surface texture of the OS (Section 3.2), as well as the IS (Section 3.3), shaped with an AWJ, using surface microtopographies measured with an optical method using the Talysurf CLI 2000 multisensory optical profilometer (Taylor-Hobson, Leicester, Great Britain) and SEM-micrographs obtained by a Quanta 200 Mark II SEM microscope (FEI Company, Hillsboro, OR, USA).

### 3.1. Study of the Influence of the Curvature of the Cut Out Shape on the IS and OS Surface Texture

**Figure 5:**
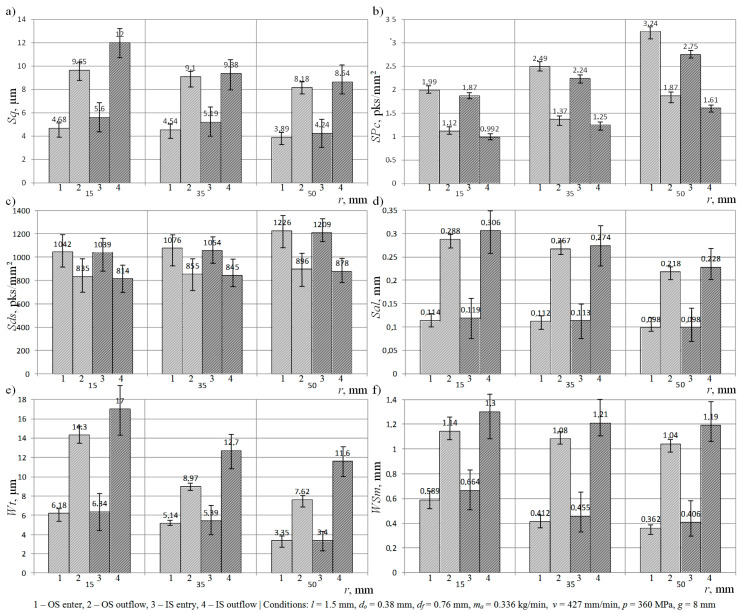
Collection of selected results of experimental studies in graphical form presenting calculated values of selected surface texture parameters using TalyMap Silver 4.1.2 software: (**a**) *Sq*; (**b**) *SPc*; (**c**) *Sds*; (**d**) *Sal*; (**e**) *Wt*; (**f**) *WSm.*

A graphical interpretation of the results of the experimental studies obtained after data processing by TalyMap Silver 4.1.2 software, depicting the relationship between the curvature of the cut out shape and the mean square deviation of the surface *Sq*, measured for the IS and OS, is shown in Figure 5a.

Analyzing the graphs, it may be observed that an increase in the radius of the cutting head trajectory from 15 mm to 50 mm reduced the value of the *Sq* amplitude (surface) parameter by an average of 16% in the areas located in the zone where the AWJ entered the material, and by an average of 26% in the areas located in the lower part of cutting zone. At the same time, it can be seen that, for each of the considered radii of the cutting head trajectory, the *Sq* amplitude (surface) parameter took on higher values in the case of measurements of the OS. For example, when the radius *r* = 15 mm, the mean square deviation of the surface, measured at the lower part of outer surface of the cutting kerf, exceeded by 2.35 μm the value of the *Sq* amplitude (surface) parameter, determined in the same area on the inner surface.

The results of experimental studies on the influence of the changes in the radius of the trajectory of the cutting head (*r*) on the arithmetic mean peak curvature (*SPc*) presented in graphical form (Figure 5b) indicate the existence of significant interdependencies between the considered parameters. The change in the curvature of the cut out shape from a value of *r* = 15 mm to *r* = 50 mm caused the *SPc* parameter to increase by 1.25 pks/mm^2^ (for IS entry). Additionally, analyzing the above graph, it may be seen that the considered surface texture parameter assumed the highest values when measurements were taken for the upper IS (*SPc* = 3.24 pks/mm^2^). However, the smallest value of the arithmetic mean peak curvature was observed when measuring the areas located on the lower OS (*SPc* = 0.992 pks/mm^2^). In addition, the obtained results indicate that, changing the radius of the cutting head trajectory, the *SPc* feature (surface) parameter adopted higher values in the areas located on the IS.

In Figure 5c, the results obtained for the *Sds* areal (surface) parameter are presented. They indicate that increasing the radius of the cutting head trajectory during the cutting process increased the density of the summits of surface irregularities for the OS and IS. At the same time, when analyzing the results of measurements of the *Sds* areal (surface) parameter, carried out for both surfaces of the cutting kerf, it may be noted that a relatively higher value of density of summits occurred on its inner surface.

The results of the influence of the radius of the curvature on the fastest decay autocorrelation length (*Sal*) are presented in Figure 5d. Analyzing the graph, it may be concluded that the increase in the radius of the trajectory of the cutting head reduced the value of this parameter for both surfaces of the cutting kerf. The presented results of the measurements also indicate that the analyzed spatial (surface) parameter reached the maximum value when measuring the lower part of the OS (*Sal* = 0.306 mm), whereas its minimum value was observed when the AWJ entered the material (IS) (*Sal* = 0.098 mm).

Figure 5e–f present the results of the experimental studies on the influence of the curvature of the shape cut out with the AWJ on the maximum height of waviness profile (*Wt*) and mean width of profile elements, within a sampling length (*WSm*). Analyzing the graph, in the case of the first waviness parameter (Figure 5e), it may be observed that the change in the radius trajectory of the cutting head from *r* = 50 mm to *r* = 15 mm influenced an increase in the value of the *Wt* parameter up to 17 μm (OS outflow), which qualified the obtained surface as an inferior quality class. At the same time, the presented results of measurements for the *Wt* waviness parameter prove that the surface texture of the OS was characterized by poorer technological quality compared with the IS. Analyzing the obtained experimental results for the mean width of profile elements within a sampling length, it may be observed that the reduction of the radius of the curvature of the shape cut out by the AWJ from a value of *r* = 50 mm to *r* = 15 mm caused an increase in the average interval of the waviness profile. In addition, the obtained results indicate that, in this situation, when the changing of the cutting head trajectory radius occurred, the *WSm* waviness parameter assumed higher values in the areas located on the outer surface of the cutting kerf.

Analyzing the obtained results of the experimental studies on the influence of the curvature of the cut out shape on selected parameters of the surface texture of the IS and OS, it may be stated that a change in the radius of the trajectory of the cutting head by over a factor of three from *r* = 50 mm to *r* = 15 mm caused an increase in the value of surface texture parameters such as Sq, Sal, Wt, and *WSm*. The most important of these parameters (*Sq* and *Wt*) undergo unfavorable changes, reaching even more than 40% of the value. The principle analyzed here also functions analogically in the opposite direction. Thus, it becomes obvious that the increase in the value of the curvature of the shape cut out by an AWJ leads to an improvement in the quality of the cutting process. In addition, it should also be noted that there is a positive influence of such a change on the increase in the arithmetic mean peak curvature (*SPc*) and the density of summits (*Sds*).

Clear qualitative differences may be observed between the surfaces of material cut by an AWJ. The values of amplitude (surface) and waviness parameters, measured on the inner and outer surfaces of cutting kerf, indicate that the surface texture of the former was characterized by a much higher quality. The differences occurring reached a level of about 20%.

On the basis of the obtained experimental results, a mathematical model, which allows for predicting values of the *Rq* roughness (profile) parameter using information about the location of the considered area (*A*_1_ and *A*_2_) depending on cutting kerf and radius values of the trajectory of the cutting head *r,* was developed. This model is given by the following dependence:

*Rq* = (−0.01158 − 0.03645γ_1_ − 0.03975γ_2_)r + 4.65903 + 2.04845γ_1_ + 6.12725γ_2_. (1)

The estimation error of the developed mathematical model, in relation to the experimental results, was relatively low and did not exceed 5%. The values of coefficients γ_1_ and γ_2_ are given in Table 4.

### 3.2. The Shaping Quality of Inner Surface of Cutting Kerf

In practice, the influence of the curvature of the cut out shape on the quality of the inner surface of the cutting kerf corresponds to the formation of cylindrical surfaces by the AWJ (the cutting of cylinders). In order to carry out their qualitative assessment, an analysis of the surface texture of areas adjacent to the upper IS (smooth and regular) and to the lower IS (more ridged and jagged) should be carried out.

**Figure 6:**
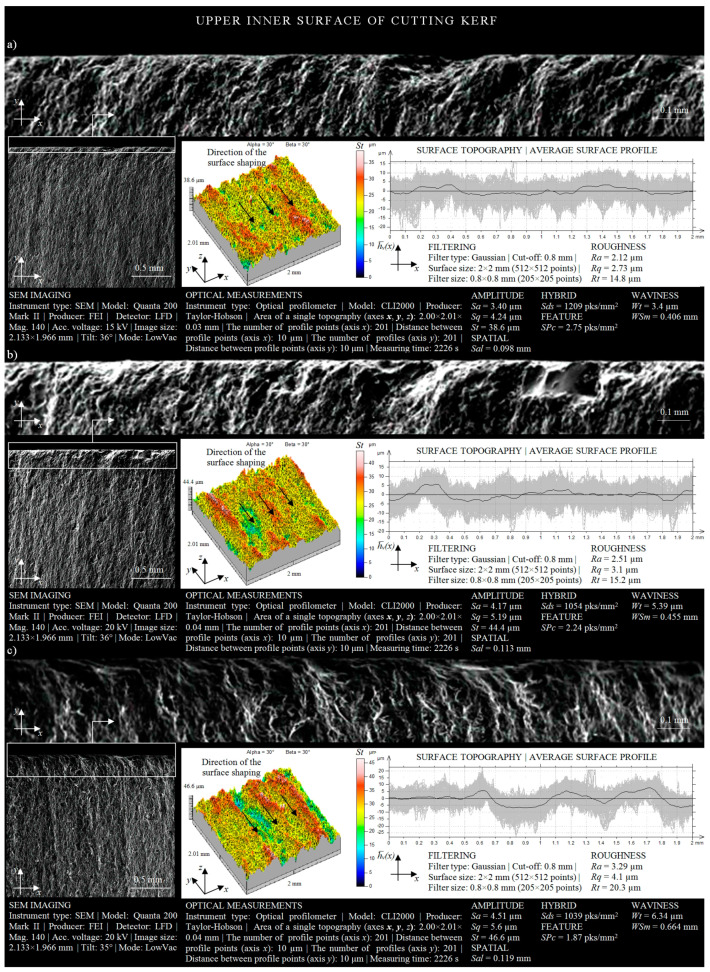
Collection of selected results of experimental studies obtained for the upper IS in a situation when the AWJ entered the sample surface (*l* = 1.5 mm, *d_o_* = 0.38 mm, *d_f_* = 0.76 mm, *m_a_* = 0.336 kg/min, *v* = 427 mm/min, *p* = 360 MPa, *g* = 8 mm): (**a**) *r* = 50 mm; (**b**) *r* = 35 mm; (**c**) *r* = 15 mm.

The surface texture of the inner surface of the cutting kerf, being the result of the influence of the machining conditions (*r* = 50 mm) when the AWJ enters the material, is presented in Figure 6a. The height analysis of the surface texture allows one to state that the total height of the surface irregularities was *St* = 38.6 μm. Approximately 70% of the analyzed cut out surface occupied areas were located at heights from 15 μm to 25 μm (yellow and green). Irregularities of a height exceeding 25 μm (red) occurred only on about 30% of the inner surface of the cutting kerf. Similar conclusions can be obtained by carrying out an analysis of the amplitude of the averaged waviness profile. As shown in the lower part of Figure 6a, the areas located near the average line of the waviness profile played an important role in its formation.

A graphical form of irregularities of the inner surface of the cutting kerf, located in the upper part of the IS, is shown in Figure 6b. Analyzing its spatial form, it may be seen that the total height of the cut out surface had a value of over 44 μm. The shaping of the surface topography presented in this image also points to the fact that the percentage of areas whose height exceeded 30 μm (red) was about 40% of the total area. In addition, by analyzing the shape of the averaged waviness profile of the IS, a locally increasing waviness may be noted whose maximum increase above the mean line was over 5 μm.

In Figure 6c, the surface texture of the upper IS, created as a result of the influence of the machining conditions (*r* = 15 mm) on the material being cut, is presented. Analyzing its geometrical shape, a large difference in the height of the irregularities (*St* = 46.6 μm) measured between the highest peak and the lowest valley can be noted. In addition, the maximum increase in the average waviness profile above the average line exceeded 8 μm, while the minimum decrease thereof below the average value was about 7 μm.

In Figure 6, the shape of the inner surface of the cutting kerf, occurring in a situation when the AWJ entered the material, is presented. Upon analyzing the obtained changes in the experimental study’s results caused by the modification of the curvature of the cut shape from *r* = 50 mm to *r* = 15 mm, visible differences in the height of the surface texture could be observed. These differences testify to the decreasing effectiveness of the influence of the AWJ on the cutting surface.

**Figure 7:**
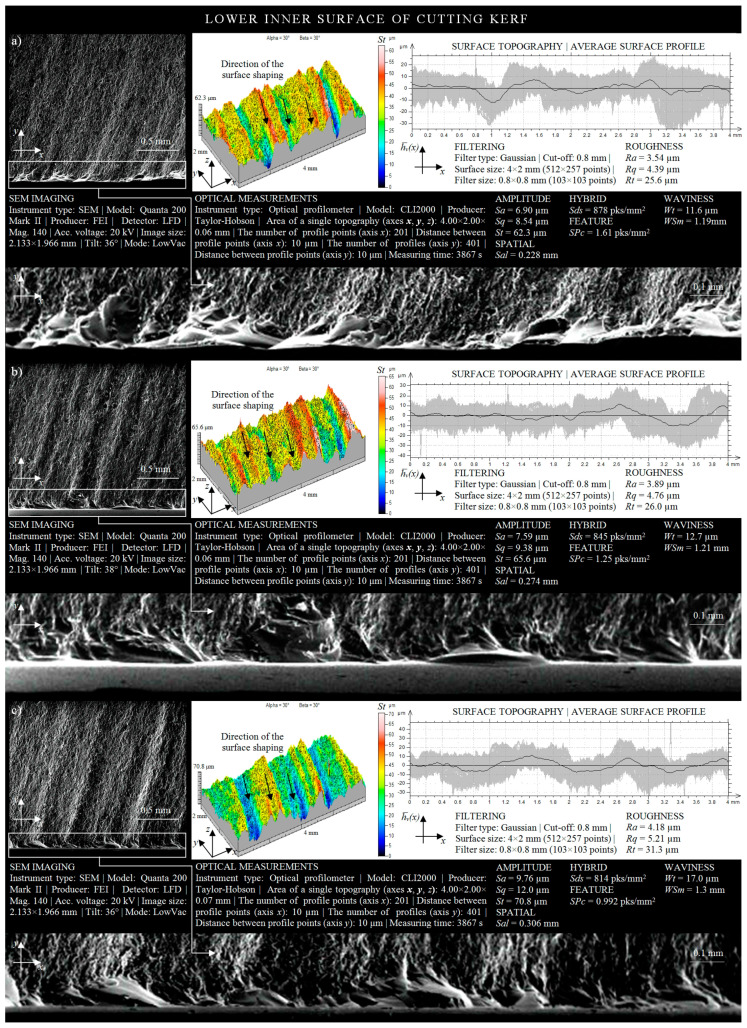
Collection of selected results of experimental studies obtained for the lower IS in a situation where the AWJ entered the sample surface (*l* = 1.5 mm, *d_o_* = 0.38 mm, *d_f_* = 0.76 mm, *m_a_* = 0.336 kg/min, *v* = 427 mm/min, *p* = 360 MPa, *g* = 8 mm): (**a**) *r* = 50 mm; (**b**) *r* = 35 mm; (**c**) *r* = 15 mm.

A graphical form of the irregularities of the inner surface of the cutting kerf, located in the lower part of the IS, is shown in Figure 7a. Analyzing its irregularities, resulting from the effect of machining conditions for *r* = 50 mm on the material being cut, it was possible to extract the profiles of slightly curved machining marks (striation). These marks were created as a result of the loss of a part of the energy produced by abrasive particles during the penetration of the AWJ into the material. The difference in the height occurring between the highest peak and lowest valley, in the case of the considered surface irregularities, was 62.3 μm. Geometric shaping of the inner surface of the cutting kerf, located in the lower part of the inner surface, indicates the fact that approximately 50% of its area was characterized by surface irregularities in levels of more than 30 μm. At the same time, significant local deviations in the amplitude of the averaged waviness profile of the analyzed surface can be observed. These deviations oscillated between the limit values of −12 μm and 8 μm.

In Figure 7b, the surface texture of the inner surface of the cutting kerf adjacent to the lower kerf, created as a result of the influence of the machining conditions (*r* = 35 mm) on the material being cut, is presented. Analyzing the surface texture of the cut out surface, it may be concluded that the total height of its irregularities was 65.6 μm. In addition, in about 80% of the area considered, the height of irregularities exceeded 30 μm. Moreover, the maximum increase in the average waviness profile above the average line was approximately 11 μm, while the minimum decrease thereof below the average value was about 9 μm.

Geometric shaping of the lower IS, resulting from the machining conditions (*r* = 15 mm) on the material being cut, is presented in Figure 7c. Analyzing the obtained microstructure of the cut out surface, one may observe an increased intensity in the machining marks appearing on it, which directly influenced the increase of both roughness and waviness of the area considered. In consequence, the height difference between the highest peak and the lowest valley was over 70 μm.

The amplitude of the averaged waviness profile of the inner surface of the cutting kerf covered, within its range (from 11 μm to −9 μm), up to six limited increases, uniformly distributed over the entire analyzed surface. Analyzing the results of the experimental studies on the influence of the trajectory of the cutting head on the roughness and waviness of the areas located in the lower surface of the cutting kerf (Figure 7), a clear influence of the curvature of the shape cut out by the AWJ on the shaping of surface texture of the analyzed surface can be observed.

Changing the radius from *r* = 50 mm to *r* = 15 mm, while maintaining the remaining parameters of the cutting process at a constant level, caused an increase in the height of irregularities (given by the *St* amplitude parameter) of 8.5 μm. A relatively high number of local increases of the amplitude of the averaged waviness profile is also observed. A deterioration in the quality of the IS, resulting from the reduction of the radius of the cutting head trajectory *r*, is also evidenced by the clear differences in its shaping, observed on SEM micrographs of the microstructure of the surface under consideration (Figure 7).

From an analysis of the height of the irregularities of the inner surface of the cutting kerf, which occurred in the zone of AWJ entry and outflow, it may be observed that the areas located in the upper part of the cutting zone were characterized by a higher quality. This fact is also confirmed by surface topographies, the amplitudes of averaged waviness profiles, and the SEM micrographs of areas adjacent to two opposite kerfs of the cut. Differences in the height of irregularities (given by *St* amplitude parameter), determined on the basis of the obtained results of surface texture measurements of areas located in the upper and lower part of the cutting zone, were as follows: 23.7 μm for *r* = 50 mm; 21.2 μm for *r* = 35 mm; 24.2 μm for *r* = 15 mm.

### 3.3. Shaping Quality of Outer Surface of Cutting Kerf

The influence of the radius of the cutting head trajectory on the quality of the OS concerns the shaping of cylindrical surfaces (holes) which may have a different diameter. In their qualitative assessment, as in the case of the cylindrical surfaces presented in Section 3.2, the analysis of the geometrical shaping of areas adjacent to two opposite surfaces of the cutting kerf is particularly important.

**Figure 8:**
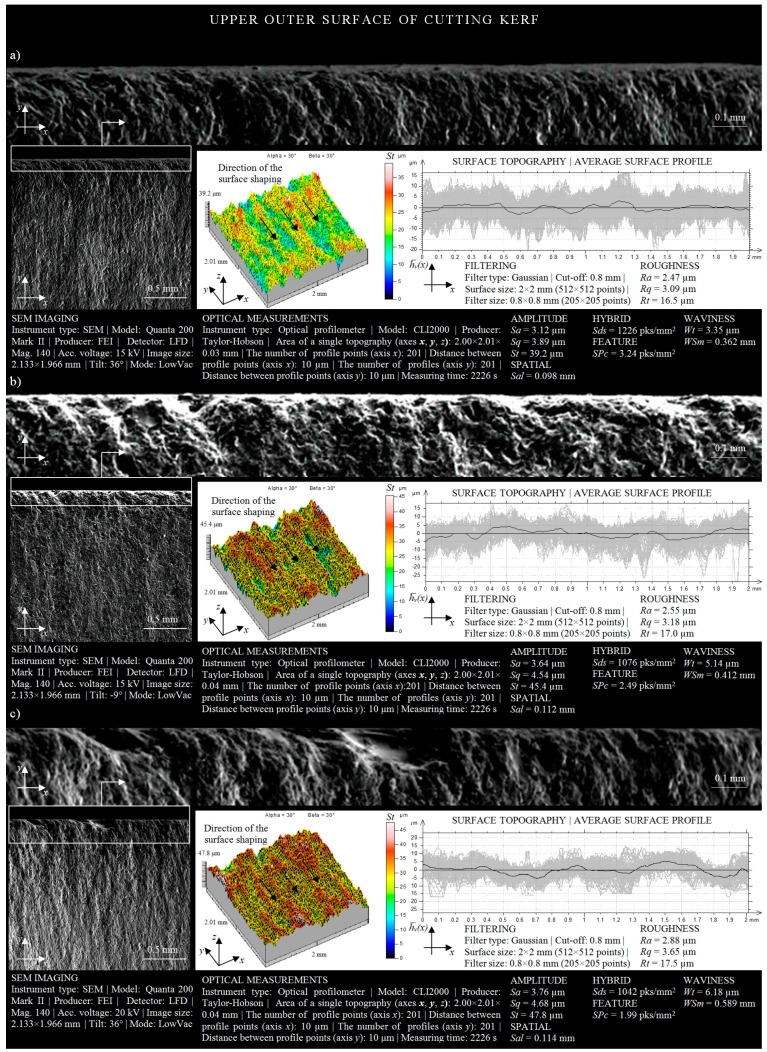
Collection of selected results of experimental studies obtained for the upper OS in a situation where the AWJ entered the sample surface (*l* = 1.5 mm, *d_o_* = 0.38 mm, *d_f_* = 0.76 mm, *m_a_* = 0.336 kg/min, *v* = 427 mm/min, *p* = 360 MPa, *g* = 8 mm): (**a**) *r* = 50 mm; (**b**) *r* = 35 mm; (**c**) *r* = 15 mm.

The surface texture of the outer surface of the cutting kerf, formed by the AWJ, is presented in Figure 8a. Analyzing its geometrical shaping, resulting from the influence of the cutting process parameters with the material being processed (*r* = 50 mm), it may be observed that the highest peak of irregularities (given by *St* amplitude parameter) had a value of 39.2 μm. Subsequent SEM micrographs and surface topographies (Figure 8b) also present the geometric shaping of the upper area of the OS.

When analyzing the obtained surface topography, one can notice that the change in the radius of the cutting head trajectory from *r* = 50 mm to *r* = 35 mm caused an increase in the maximum height of the peaks appearing on it up to the value of 45.4 μm. In addition, surface irregularities with a height exceeding 30 μm occurred on 50% of the analyzed area of the OS. Considering the averaged surface waviness profile presented in Figure 8b, it may be stated that its maximum increase above the mean line reached a height of 4 μm, while the minimum decrease was of −4 μm.

The texture of the surface located in the upper area of the outer surface of the cutting kerf, which was the result of the machining conditions (*r* = 15 mm) on the material being cut, is presented in Figure 8c. Analyzing its spatial form, one may observe a large variation in the heights of irregularities occurring between the highest peak and the deepest valley. The maximum height of the surface microstructure (given by *St* amplitude parameter) was approximately 47.8 μm. In addition, the geometrical shaping of the upper area of the outer surface of the cutting kerf indicates that approximately 80% of its irregularities were characterized by a height whose value was more than 30 μm. However, the height of amplitude of the average waviness profile presented in Figure 8c oscillated between two extreme values: −5 μm and 5 μm.

Analyzing the results of experimental studies on the influence of the curvature of the shape cut out by AWJ on the surface texture of areas located in the upper part of the outer surface of the cutting kerf (Figure 8), it may be concluded that the change in the trajectory of the cutting head from *r* = 15 mm to *r* = 50 mm caused a reduction in surface irregularities in the area considered by 8.6 μm. At the same time, the values of the average waviness profile $\bar{h_{v}}$*(x)* decreased by approximately 2 μm. Differentiations in roughness and waviness of the OS, caused by the increase in the radius of the trajectory of the cutting head during the cutting process, were also revealed by the SEM micrographs of the microstructure presented in Figure 8.

**Figure 9:**
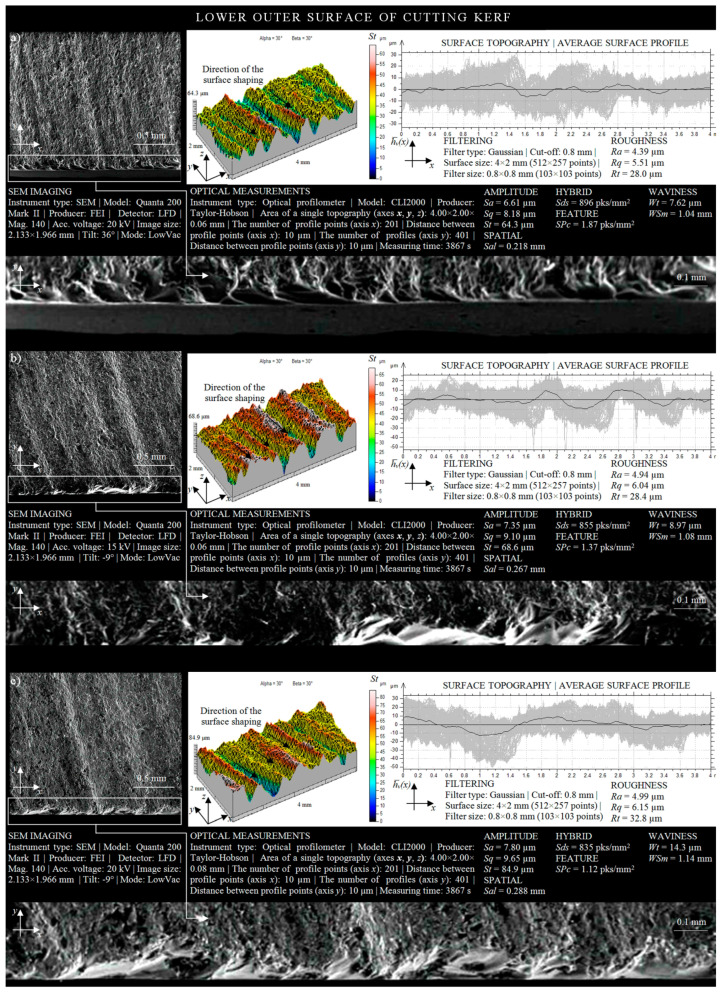
Collection of selected results of experimental studies obtained for the lower OS in a situation where the AWJ entered the sample surface (*l* = 1.5 mm, *d_o_* = 0.38 mm, *d_f_* = 0.76 mm, *m_a_* = 0.336 kg/min, *v* = 427 mm/min, *p* = 360 MPa, *g* = 8 mm): (**a**) *r* = 50 mm; (**b**) *r* = 35 mm; (**c**) *r* = 15 mm.

In Figure 9, the geometrical shaping of the lower areas of the OS is presented. The surface texture, formed as a result of the effect of the influence of the curvature of the cut shape *r* = 50 mm on the material being processed is shown in Figure 9a. Analyzing the shaping of the area located in the more sensitive zone of AWJ outflow, it is possible to discern the outlines of machining marks (striation) that arose as a result of the AWJ losing some of its energy during removal of the upper surface of the material being cut. The total height of the peaks obtained in this way in the microstructure of the OS (given by *St* amplitude parameter) was 64.3 μm. About 20% of the analyzed area had surface irregularities, the height of which exceeded 45 μm. The amplitude of the averaged waviness profile of the outer surface of the cutting kerf $\bar{h_{v}}$*(x)* oscillated between two extreme values of −6 μm and 5 μm in relation to the mean line. 

The texture of the surface located in the lower area of the outer surface of the cutting kerf is presented in a graphical form in Figure 9b. Analyzing its shaping, it may be observed that the change in the radius of the curvature of the cut out shape from *r* = 50 mm to *r* = 35 mm caused an increase in the highest peak of the irregularities to a height of over 68 μm. However, when analyzing the surface topography of the considered area occurring in the lower part of the outer surface of the cutting kerf, it may be concluded that the percentage of surface irregularities whose height exceeded 45 μm (red) was about 50% of the average waviness profile $\bar{h_{v}}$*(x)*, which covered the range from −10 μm to 10 μm.

The surface texture of the area of the OS, adjacent to the lower surface of the cutting kerf, is presented in Figure 9c. Considering its geometrical shaping, resulting from the influence of the process parameter *r* = 15 mm on the material being processed, one may clearly observe the machining marks created as a result of the interaction of the AWJ on the material being processed. In addition, the highest peak of irregularities of the analyzed cut surface occurred at a height (given by *St* amplitude parameter) of 84.9 μm at the averaged waviness profile contained in ranges from about −12 μm to 10 μm relative to the mean line.

Analyzing the shaping of the areas occurring in the lower part of the OS, as presented in Figure 9, resulting from the curvature of the cut out shape, it may be concluded that the change in the radius of the cutting head trajectory from *r* = 15 mm to *r* = 50 mm reduced surface irregularities of 20.5 μm. It also changed the average waviness profile $\bar{h_{v}}$*(x)*, whose amplitude decreased with the increase in the curvature of the shape cut out by the AWJ. Differences in roughness and waviness occurring in the areas located in the lower part of the outer surface of the cutting kerf, caused by the change in the radius of the cutting head trajectory, are clearly observed in the SEM-micrographs. The increase in the curvature of the cut out shape (*r*) during the AWJ cutting process improved the quality of the outer cut surface by limiting the intensity of the occurrence of striation on it.

Comparing the height of the areas of the external surface of the cutting kerf located in the zone of AWJ entry and outflow from the material, it may be concluded that the areas adjacent to the upper cutting kerfs were characterized by a relatively better technological quality. The sample SEM micrographs, surface topographies, and averaged waviness profiles presented in Figure 8 and Figure 9 provide evidence for the information given above. Differences in the height of the irregularities (given by *St* amplitude parameter), determined on the basis of the surface texture measurements carried out for areas located in the upper and lower part of the cutting zone, were as follows: 23.7 μm for *r* = 50 mm; 23.2 μm for *r* = 35 mm; 37.1 μm for *r* = 15 mm.

Analyzing the shaping of the surface texture of areas located in the upper part of the inner and outer surface of the material cut out by the AWJ (Figure 6 and Figure 8), it may be observed that the outer surfaces were characterized by much worse technological quality in comparison with inner surfaces. Differences in the height of irregularities (given by *St* amplitude parameter), determined on the basis of the obtained results of measurements of both surfaces of the cutting kerf, were as follows: Δ*_Str = 50_* = 0.6 µm; Δ*_Str = 35_* = 1 µm; Δ*_Str = 15_* = 1.3 µm.

When comparing the obtained values of the amplitude (surface) parameter *St*, measured in areas located in the more sensitive outflow zone of the AWJ for both cut out surfaces of the lower IS and lower OS (Figure 7 and Figure 9), it may be clearly observed once again that the height of irregularities of the outer surface were higher. Differences in the heights of irregularities measured for the inner and outer surfaces of the cutting kerf were as follows: Δ*_Str = 50_* = 2.1 µm; Δ*_Str = 35_* = 3 µm; Δ*_Str = 15_* = 14.1 µm.

## 4. Conclusions

The curvature of a shape cut out by an AWJ, resulting from the size of the radius of the cutting head trajectory, is one of the key requirements necessary for ensuring the required surface quality of materials shaped by AWJ machining. An important goal of the experimental studies carried out and presented in this work was to determine its influence on the quality of the inner and outer surfaces of the cutting kerf. This goal was accomplished by cutting the shape of a spiral. In such a form, the sample was used in experiments during which the influence of the radius of the cutting head trajectory on selected surface texture parameters of the inner and outer surface of the cutting kerf located in the zone of AWJ entry and outflow was analyzed. The obtained results of measurements and analyses allowed one to draw the following detailed conclusions:

1. The obtained results of the experimental studies confirmed that the effect of the curvature of the cut shape is important from the point of view of the efficiency of the glass-based brittle materials cutting process using the AWJ. On the basis of the obtained experimental results, it may be concluded that the feed speed should be limited when *r* < 35 mm.
2. The determined mathematical model in Equation (1), which describes the influence of the cutting head trajectory on the surface quality of the soda–lime glass, describes with approximately 95% accuracy the relationships occurring between the trajectory radius of the cutting head and the amplitude *Sq* (surface) parameter. This means that the model was adequate for the experimental data and could be successfully used to predict the quality of both surfaces of the cutting kerf. The model ran properly in the range of radius variation *r* = 15–50 mm.
3. The determined values of the surface texture parameters for the inner and outer surfaces of cutting kerf (Figure 5, Figure 6, Figure 7, Figure 8 and Figure 9) clearly indicate that these surfaces were characterized by worse technological quality than cylindrical surfaces. The maximum difference in the total height of the surface (*St*) existing between the considered surfaces (for *r* = 15 mm) was almost 20%, which should be a sufficient condition for planning cutting operations, so that the workpiece is shaped mainly by internal surfaces.
4. The results of experimental studies presented in this article do not exhaust all the issues related to the problem of curvilinear cutting of brittle materials (glass) using AWJ, particularly the aspects of their surface quality inspection. The authors see a strong need to continue this interesting and promising subject, especially in the context of AWJ process optimization for industrial applications planning subsequent publications in this area in the near future.

## Figures and Tables

**Table 1:** Characteristics of roughness and waviness parameters calculated during experimental studies.

Group of Parameters	Symbol	Unit	Description
Roughness (profile)	*Ra*	µm	Arithmetical mean deviation of the roughness profile
	*Rq*	µm	Root-mean-square deviation of the roughness profile
	*Rt*	µm	Total height of the profile on the evaluation length
Amplitude (surface)	*Sa*	µm	Arithmetic mean deviation of the surface
	*Sq*	µm	Root-mean-square (RMS) deviation of the surface
	*St*	µm	Total height of the surface
Spatial (surface)	*Sal*	mm	Fastest decay autocorrelation length
Areal (surface) ^1^	*Sds*	pks/mm^2^	Density of summits
Feature (surface)	*SPc*	pks/mm^2^	Arithmetic mean peak curvature
Waviness (profile)	*Wt*	µm	Maximum height of waviness profile
	*WSm*	mm	Mean width of profile elements, within a sampling length

^1^ As reported by Blateyron [39], the *Sds* parameter corresponds to *Spd* in ISO 25178-2, but the discrimination method is different.

**Table 2:** General characteristics of soda–lime glass.

**Chemical Composition**													
SiO_2_, %	Na_2_O, %			CaO, %		MgO, %	Al_2_O_3_, %	K_2_O, %			SO_2_, %		Fe_2_0_3_, %
72.60	13.90			8.40		3.90	1.10	0.60			0.20		0.11
**Strength**													
Flexural							Compressive						
Annealed, MPa		Heat-strengthened, MPa			Toughened, MPa		Annealed, MPa		Heat-strengthened, MPa			Toughened, MPa	
41		83			165		19		39			77	
**Physical Properties**													
Density, kg/m^3 1^		Mohs hardness			Modulus of elasticity, GPa		Shear modulus, GPa		Poisson’s ratio			Coeff. of thermal stress, MPa/°C	
2500		5–6			72		30		0.23			0.62	
Thermal conductivity, W/m·K		Specific heat, kJ/kg·K			Coeff. of linear expansion, °C		Index of refraction ^2^		Softening point, °C			Annealing point, °C	
0.937		0.88			8.3 × 10^−6^		1.5		715			548	
Max. working temperature, °C							Thermal shock Δ, °C						
Not Toughened			Toughened				Not Toughened			Toughened			
110			150				50			118			

^1^ At 18 °C; ^2^ in visible wavelength range *λ* = 380–780 nm.

**Table 3:** Characteristics of observation/measurement systems used in experimental studies.

No.	Instrument Type	Model	Producer	Configuration and Features
1.	Multisensory optical profilometer	CLI2000	Taylor-Hobson (Leicester, Great Britain)	Components: laser triangulation sensor LK-031 (Keyence Corp., Osaka, Japan) Features (sensor): scanning frequency: 2000 Hz, measuring range: 10 mm, resolution: 1 μm (vertical), 30 µm (lateral), measuring slope: 40°, speed: 30 mm/s Features (instrument): measuring capacity: 200 × 200 × 200 mm, axis traverse length: 200 mm, axis resolution: 0.5 μm, dimensions: 800 × 800 × 800 mm, measuring speed: 0.5, 1, 5, 10, 15, and 30 mm/s, positioning speed: 30 mm/s
				Software: Talyscan CLI 2000 2.6.1+ TalyMap Silver 4.1.2 (Digital Surf, Besançon, France)
2.	SEM microscope	Quanta 200 Mark II	FEI Company, (Hillsboro, OR, USA)	Components: detectors: SEI (Everhart-Thornley SED, low-vacuum SED (LFD), gaseous SED (GSED)), BEI (solid-state (BSED), gaseous SED (GSED)), specimen stage: eucentric goniometer stage (four-axis motorized) Features: magnification range: 30 × to ~1,000,000 ×, vacuum pressure in the specimen chamber: < 0.0006 Pa (HVM), 10–130 Pa (LVM), accelerating voltage: 0.2–30 kV, resolution (using HVM): 3.0 nm at 30 kV SEI, 4.0 nm at 30 kV BSE, 10 nm at 3 kV SEI, (using LVM): 3.0 nm at 30 kV SEI, 4.0 nm at 30 kV BSE, < 12 nm at 3 kV SEI
				Software: dedicated FEI software

**Table 4:** Method for designation of measured area on kerf cutting.

Area location	γ_1_	γ_2_
IS enter	1	0
IS outflow	1	1
OS enter	0	0
OS outflow	0	1

## Nomenclature

AWJ	Abrasive water jet
BEI	Backscattered electron imaging
BSED	Solid-state secondary electron detector
CAD	Computer-aided design
ESEM	Environmental scanning electron microscope
GSED	Gaseous secondary electron detector
IS	Inner surface of cutting kerf
LFD	Low-vacuum secondary electron detector
OS	Outer surface of cutting kerf
RMS	Root mean square
SED	Secondary electron detector
SEI	Secondary electron image
SEM	Scanning electron microscope
*df*	Focusing tube diameter, mm
*do*	Water jet orifice diameter, mm
*g*	Material thickness, mm
$\bar{h_{v}}$ *(x)*	Average waviness profile, μm
*l*	Standoff distance, mm
*ma*	Abrasive feed rate, kg/min
*n*	Jet lag distance, mm
*p*	Water jet pressure, MPa
*pmax*	Nominal pressure, MPa
*r*	Machining radius, mm
*v*	Traverse speed, mm/min
*Qmax*	Maximum water flow rate, dm^3^/min
*Ra*	Arithmetical mean deviation of the roughness profile, μm
*Rq*	Root-mean-square deviation of the roughness profile, μm
*Rt*	Total height of the profile on the evaluation length, μm
*Sa*	Arithmetic mean deviation of the surface, μm
*Sal*	Fastest decay autocorrelation length, mm
*Sds*	Density of summits of the surface, pks/mm^2^
*SPc*	Arithmetic mean peak curvature, pks/mm^2^
*Sq*	Root-mean-square deviation of the surface, μm
*St*	Total height of the surface, μm
*Ua*	Accelerating voltage, kV
*WSm*	Mean width of profile elements, within a sampling length, mm
*Wt*	Maximum height of waviness profile, μm
